# Elastin-Plasma Hybrid Hydrogels for Skin Tissue Engineering

**DOI:** 10.3390/polym13132114

**Published:** 2021-06-28

**Authors:** Marija Stojic, Joaquín Ródenas-Rochina, María Luisa López-Donaire, Israel González de Torre, Miguel González Pérez, José Carlos Rodríguez-Cabello, Lucy Vojtová, José Luis Jorcano, Diego Velasco

**Affiliations:** 1 CEITEC-Central European Institute of Technology, Brno University of Technology, Purkyňova 123, 61200 Brno, Czech Republic; marija.stojic@ceitec.vutbr.cz (M.S.); lucy.vojtova@ceitec.vutbr.cz (L.V.); 2 Department of Bioengineering & Aerospace Engineering, University Carlos III of Madrid (UC3M), Avenida Universidad 30, 28911 Leganés, Spain; marisalopezdonaire55@gmail.com (M.L.L.-D.); jjorcano@ing.uc3m.es (J.L.J.); 3 Centre for Biomaterials and Tissue Engineering, CBIT, Universitat Politecnica de Valencia, Camino de Vera s/n., 46022 Valencia, Spain; jrodenasr@gmail.com; 4 BIOFORGE (Group for Advanced Materials and Nanobiotechnology), University of Valladolid, CIBER-BBN, 47011 Valladolid, Spain; mgonzalezperez@bioforge.uva.es (M.G.P.); roca@bioforge.uva.es (J.C.R.-C.); 5 Instituto de Investigación Sanitaria Gregorio Marañón, 28007 Madrid, Spain

**Keywords:** bilayered in vitro skin substitutes, elastin like recombinamers, human plasma-derived fibrin hydrogels, fibrin hydrogels, hybrid plasma-elastin hydrogels, bioengineered skin, skin tissue engineering

## Abstract

Dermo-epidermal equivalents based on plasma-derived fibrin hydrogels have been extensively studied for skin engineering. However, they showed rapid degradation and contraction over time and low mechanical properties which limit their reproducibility and lifespan. In order to achieve better mechanical properties, elasticity and biological properties, we incorporated a elastin-like recombinamer (ELR) network, based on two types of ELR, one modified with azide (SKS-N_3_) and other with cyclooctyne (SKS-Cyclo) chemical groups at molar ratio 1:1 at three different SKS (serine-lysine-serine sequence) concentrations (1, 3, and 5 wt.%), into plasma-derived fibrin hydrogels. Our results showed a decrease in gelation time and contraction, both in the absence and presence of the encapsulated human primary fibroblasts (hFBs), higher mechanical properties and increase in elasticity when SKSs content is equal or higher than 3%. However, hFBs proliferation showed an improvement when the lowest SKS content (1 wt.%) was used but started decreasing when increasing SKS concentration at day 14 with respect to the plasma control. Proliferation of human primary keratinocytes (hKCs) seeded on top of the hybrid-plasma hydrogels containing 1 and 3% of SKS showed no differences to plasma control and an increase in hKCs proliferation was observed for hybrid-plasma hydrogels containing 5 wt.% of SKS. These promising results showed the need to achieve a balance between the reduced contraction, the better mechanical properties and biological properties and indicate the potential of using this type of hydrogel as a testing platform for pharmaceutical products and cosmetics, and future work will elucidate their potential.

## 1. Introduction

Dermo-epidermal equivalents based on plasma-derived fibrin hydrogels offer a promising approach to skin engineering since they allow the preparation of more sophisticated laboratory-grown skin substitutes which better resemble the structure and function of human skin [1,2,3,4,5,6]. They also allow an efficient production of natural collagen by human fibroblasts and a fully autologous process for skin grafting [2,7,8,9]. Besides these properties, the presence of a complex mixture of plasma proteins, lipids and multiple platelet growth factors that are physically entrapped in the plasma-derived fibrin matrix once it is formed [10], helps to provide a more suitable 3D environment to promote migration, proliferation and differentiation of the cells in the wound bed [2,11,12,13]. There are also important proteins in plasma such as galectin-3 and interleukin (IL)-6 that are implicated in several processes central to the wound-healing response, specifically in the regulation of inflammation, activation, and proliferation of leukocytes, endothelial cells, keratinocytes, and fibroblasts, with these types of protein being also used as prognostic biomarkers [14,15,16,17]. However, current drawbacks of these types of hydrogel lies not only in their moderate tensile strength, but they also suffer a rapid degradation and contraction over time, limiting their reproducibility and lifespan [18,19,20]. PEGylation of the fibrinogen contained in human plasma or the combination with natural polymeric networks, such as alginate or agarose [21,22,23,24,25,26], have been utilized to overcome the aforementioned limitations. Another promising strategy could be the preparation of interpenetrating polymer network (IPN) hydrogels based on plasma-derived fibrin and extracellular matrix (ECM) components. One lead material is elastin which has the potential to prevent skin contractures, improve scar quality and enhance elasticity of future dermal extracellular matrix (ECM) [27,28]. Elastin is a vital protein component of the ECM present in many mammalian tissues, which require elasticity as part of their function. In this sense, elastic fibers represent 2–4% of the total dry weight of the dermis in adults [29]. They are composed of two morphologically and chemically distinctive components: (i) elastin, connective tissue protein, which makes approximately 90% of elastic fiber, and (ii) microfibrils which intersperse and surround elastin [30]. Mature elastin is an insoluble polymer composed of several tropoelastin molecules covalently bound to each other by cross-links. The nature of elastin itself has hindered the study of its properties and structure, mainly due to its insolubility in water and backbone mobility. In this sense, elastin-like recombinamers (ELRs) are genetically engineered biomaterials based on the repetition of the pentamer found in natural elastin (VPGVG) [31] where V stand for L-valine, P for L-proline, G for glycine. This sequence is the main responsible of the elastic properties of elastin. In this pentapeptide, the fourth amino acid can be changed for any amino acid except L-proline [32]. The use of recombinant DNA technologies gives us precise control over ELRs amino acid sequences, chain length and the possibility of incorporating bioactive sequences [33] or specific residues in concrete positions in order to modulate the physical and chemical properties of these ELRs. They exhibit extraordinary properties which mimic certain sequences of natural elastin and make them desirable in the fields of tissue engineering and regenerative medicine [34,35,36,37,38,39,40,41,42,43,44].

Although 2D elastin-based scaffolds and fibroblasts is a well explored pairing, 3D-based scaffolds with embedded fibroblasts are not so extensively explored [45,46,47,48,49]. One of the main reasons could be the use of non-cytocompatible conditions to crosslink elastin derivatives, mostly due to the use of crosslinkers such as glutaraldehyde or dimethyl isocyanate that are cytotoxic or need cytotoxic conditions [50,51]. Nevertheless, some examples can be found in literature in which fibroblast are encapsulated inside 3D ELR hydrogels that produce optimal cell behaviour and proliferation [52]. If we pay attention to other cell lines apart from fibroblasts, 3D ELR-based scaffolds embedded with different cell lines (mesenchymal stem cells, human umbilical vein endothelial cells and smooth muscle cells among others) can be found in literature in the last few years [52,53,54,55]. In this work an ELR containing both the tri-amino acid sequence, arginine-glycine-aspartate (RGD) to enhance the cell adhesion and proliferation, and serine amino acid substituting some of the valine in the fourth position of the base VPGVG pentamer will be designed and produced.

The main objective of this study is to provide elasticity and better mechanical properties to human plasma hydrogels by the incorporation of crosslinked elastin. To this aim, we incorporated a ELR network, based on two types of ELRs, one modified with azide (ELR-N_3_) and other with cyclooctyne (ELR-Cyclo) chemical groups, into a protocol developed by our group for the generation of a human plasma-derived bilayered (including dermis and epidermis) skin model. To this end, we evaluated ELR-N3 and ELR-Cyclo reacted at molar ratio 1:1 at three different ELR physiological concentrations (1, 3, and 5 wt.%) in the presence of plasma-derived fibrin hydrogels, and their physical and biological properties. We first studied the effect of incorporating ELRs into plasma-derived fibrin hydrogels via gelation time, hydrogel contraction with and without encapsulated human primary fibroblasts (hFBs), and mechanical and structural characterization. Furthermore, we studied the biological properties of the elastin-plasma hydrogels and their interaction with hFBs and human primary keratinocytes (hKCs). In particular, the proliferation of the encapsulated hFBs inside of the matrices at different time points (1, 3, 7 and 14 days) was studied. We also studied the proliferation of hKCs seeded on top of the matrices at 2 days. These dermo-epidermal equivalents with fully tunable properties could represent a new generation of in vitro skin models. 

## 2. Materials and Methods

### 2.1. Materials

Fresh frozen human platelet-poor plasma was obtained from voluntary donors of a local blood bank (Banco de Sangre del Centro Comunitario de Transfusion del Principado de Asturias (CCST)) and it was carefully handled and treated according to the standards and regulations of the American Association of Blood Banks [56]. Plasma batches were aliquoted upon arrival and stored at −80 °C until further use [57]. Tranexamic acid (AmchaFibrin) (Rottapharm SL, Barcelona, Spain) was used as received. Sodium chloride (NaCl) and calcium chloride (CaCl_2_) were purchased from Sigma Aldrich, St. Louis, MO, USA. Sterile NaCl and CaCl_2_ solution in Milli-Q ultra-pure water (Sigma Aldrich, St. Louis, MO, USA) were prepared at 0.9 wt.% and 1 wt.%, respectively and filtered through 0.22 polyethersulfone (PES) (Millipore) filters (Sigma Aldrich, St. Louis, MO, USA). Phosphate-buffered saline (PBS) solution was also purchased from Sigma Aldrich, St. Louis, MO, USA. Ethanol was purchased from Scharlab, Barcelona, Spain.

2-Azidoethyl(2,5-dioxopyrrolidin-1-yl) carbonate and (1R,8S,9S)-bicyclo [6.1.0] non-4-yn-9-ylmethyl succinimidyl carbonate were obtained from Galchimia S.L., A Coruña, Spain. Di-methyl sulfoxide (DMSO) and diethyl ether were purchased from Sigma-Aldrich (San Luis, MO, USA) and used as received.

Elastin-like recombinamer polymer (ELR) with arginine-glycine-aspartic acid (RGD) regions was purified from *Escherichia coli* following already stablished protocols developed by Cabello et al. [54] and kindly supplied by BIOFORGE at the University of Valladolid. Two different types of elastin, one modified with azide (N3) and other with cyclooctyne (Cyclo) chemical groups were used in this work.

### 2.2. Elastin-Like Recombinamer (ELR) Biosynthesis and Purification

Design, production and purification of the ELRs used in this work have been performed using standard genetic engineered recombinant techniques already described elsewhere [58]. A high transition temperature (Tt), RGD-containing ELR has been designed. This new ELR has been called SKS (serine-lysine-serine sequence) with a molecular weight of 82,487 Da and with an amino acid structure detailed below:

MESLLP [(VPGVG VPGSG VPGVG VPGKG VPGVG VPGSG VPGVG)2 VAVTGRGDSP ASSGGGGSGGGGSGGGGS (VPGVG VPGSG VPGVG VPGKG VPGVG VPGSGVPGV-G)2]6-V

Purification of SKS consisted of several cycles of temperature-dependent reversible precipitations by centrifugation, below and above their transition temperature (Tt), making use of the intrinsic thermal behavior of these compounds. The obtained ELRs were dialyzed against ultrapure deionized water (Millipore, Sigma Aldrich, St. Louis, MO, USA) and then lyophilized. The purity and molecular weight of the ELRs were verified by sodium dodecyl sulphate polyacrylamide gel electrophoresis (SDS-PAGE) and matrix assisted laser desorption/ionization time-of-flight (MALDI-TOF) mass spectrometry using a Voyager STR apparatus from Applied Biosystems, Foster City, CA, USA. Amino-acid composition analysis was performed by high-performance liquid chromatography (HPLC). Finally, additional characterization of ELRs was accomplished using Fourier-transform infrared spectroscopy (FTIR), differential scanning calorimetry (DSC) and proton nuclear magnetic resonance (1H-NMR) techniques.

### 2.3. Synthesis of Azide/Cyclooctyne-Bearing ELRs

The synthesis of azide or cyclooctyne-modified SKS was undertaken by transformation of the ε-amine group in the lateral lysine chain as follows: A solution of 2-azidoethyl (2,5-dioxopyrrolidin-1-yl) carbonate or (1R,8S,9S)-bicyclo[6.1.0]non-4-yn-9-ylmethyl succinimidyl carbonate in DMSO was added to a solution of SKS in DMSO and the resulting mixture was stirred at room temperature for 48 h. After this time, diethyl ether was added to the mixture to give a white precipitate, which was dried under reduced pressure and dissolved in ultrapure water. The aqueous solution was dialyzed against ultrapure water and lyophilized to yield a white recombinamer (SKS-azide). The reaction was designed to modify three quarters of the SKS lysine residues.

### 2.4. Preparation of Plasma-Derived Fibrin Hydrogels

For the preparation of the plasma-derived fibrin hydrogels a protocol established by Meana et al. [3] was followed. The experiments described in this article were performed using plasma from the same batch. Human plasma, with a known fibrinogen concentration, was preserved at −80 °C and defrosted in a 37 °C hot bath prior to hydrogel preparation. Hydrogels (0.813 mL volume) containing a final fibrinogen concentration of 1.2 mg/mL were prepared in a glass vial (Labbox, Barcelona, Spain) with a 19 mm inner diameter. The protocol was as follows, the required volume of initial plasma (with an initial fibrinogen concentration of 2.4 mg/mL) was added to a vial (400 μL) and subsequently supplemented with the antifibrinolitic agent, AmchaFibrin, at a final concentration of 0.008 wt.% (6.5 μL). Finally, 1 wt.% CaCl_2_, previously diluted in 0.9 wt.% NaCl solution to a 0.08 wt.% final concentration, was added (0.65 mL) to the vial in order to trigger the coagulation cascade. Between each addition, the solution was vortexed. These vials were introduced into an incubator (Sheldon Manufacturing, Inc., Cornelius, OR, USA) at 37 °C with 5% CO_2_ and 40% relative humidity and left undisturbed until gelation occurred within an hour. Once the hydrogels were formed, they were detached from the glass vials using warmed (37 °C) PBS. Hydrogels were transferred into a 35 mm diameter petri dish (P35) (Thermo Fisher Scientific, Waltham, MA, USA) in order to perform further studies.

### 2.5. Preparation of Elastin-Plasma Hydrogels

**Figure 1:**
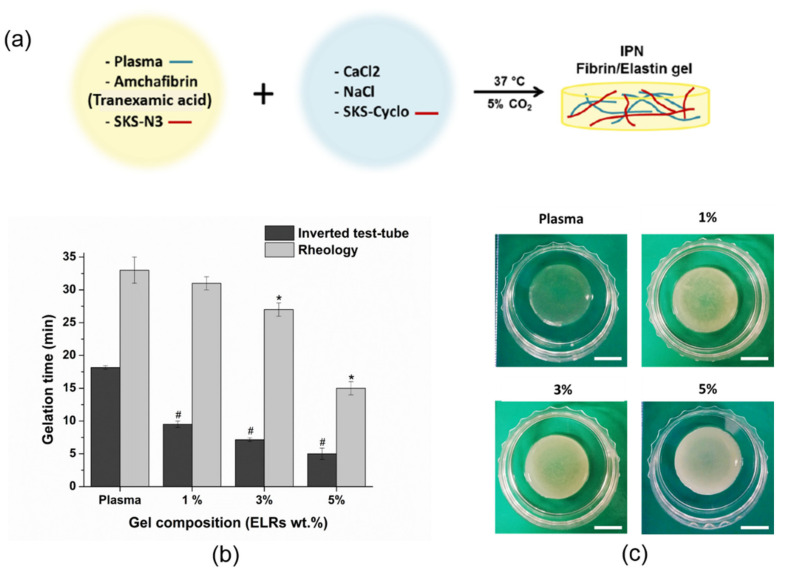
(**a**) Illustration of the components and mixture conditions for the preparation of the hybrid plasma-SKS (serine-lysine-serine sequence) hydrogels; (**b**) Gelation time at 37 °C determined by inverted test tube and rheology methods (data expressed as mean ± standard deviation (SD), n = 3) and the. Analysis of variance (ANOVA) results performed for each hydrogel with respect to plasma samples by rheology at significant level of * *p* < 0.05 and by inverted test-tube (# < 0.05). (**c**) Digital images of plasma and plasma elastin-like recombinamer polymers (ELRs) hybrid hydrogels in phosphate-buffered saline (PBS) after removal from the vial. The scale bars correspond to 1 cm. Percentages indicate % *w*/*v* of elastin content in hybrid hydrogels.

For elastin-plasma hybrid hydrogels, the procedure was similar to the plasma hydrogel preparation described in Section 2.4 but slightly modified considering the protocol carried out by González de la Torre et al. [59] for elastin recombinamer hydrogels. Thus, two solutions of Elastin-N3 (SKS-N3) and Elastin-Cyclo (SKS-Cyclo) (at the desirable concentration) were prepared separately in order to avoid elastin network formation prior to plasma coagulation. The SKS-N3 was dissolved in plasma supplemented with AmchaFibrin (0.008 wt.%) while SKS-Cyclo was dissolved in 0.9 wt.% NaCl (Figure 1a). Molar ratio of SKS-N3 and SKS-Cyclo was 1:1 and prepared solutions were kept at 4 °C overnight. Plasma-elastin hydrogels were obtained by simply mixing these two solutions at 4 °C. Elastin-plasma hybrid hydrogel was prepared with a final fibrinogen concentration of 1.2 mg/mL and the elastin recombinamer content was added at three different concentrations (1, 3, and 5 wt.%).

Hydrogel solutions were mixed in glass vials of 19 mm of inner diameter and incubated at 37 °C with 5% CO_2_ and 40% relative humidity for 1 h to allow the polymerization. Finally, hydrogels were detached from vials using warmed (37 °C) PBS and placed in a 35 mm diameter Petri dish (P35). For each concentration three replicates for each hydrogel were made.

### 2.6. Gelation Time Determination

Plasma and elastin-plasma hybrid hydrogels were prepared as previously indicated in Section 2.4 and Section 2.5 and the gelation time was studied by test tube inversion assay as reported by Vanderhooft et al. [60]. The hydrogel solution was placed into the 19 mm in diameter glass vials and introduced into the incubator under 37 °C with 5% CO_2_ and 40% relative humidity. Gelation time was checked every 30 s by slightly inverting the vial. The time at which the hydrogel was in the solid phase, staying stuck at the bottom surface of the vial, was determined as the gelation time. Three replicates of each sample were evaluated. Data were expressed as mean ± standard deviation (SD). Hydrogel gelation was also determined by rheological experiments (three replicates of each sample) which are described in the Section 2.7.2.

### 2.7. Physicochemical Properties of Hybrid Plasma-ELRs Hydrogels

#### 2.7.1. Azide/Cyclooctyne-Bearing ELRs and Hybrid Plasma-ELRs Hydrogel Characterization

Azide/cyclooctyne-bearing ELRs (SKS-N3 and SKS-Cyclo) were characterized by nuclear magnetic resonance spectroscopy (^1^H-NMR) using a Varian AV-400 (AgilentTechnologies, Santa Clara, CA, USA), Fourier-transform infrared spectroscopy (FTIR) using (Bruker Tensor 27 (Billerica, MA, USA)), differential scanning calorimetry (DSC) using a Mettler Toledo DSC822e (Columbus, OH, USA) and MALDI-TOF mass spectrometry using a Bruker DaltonicsAutoflex Speed Instrument (Bruker, Billerica, MA, USA). This characterization is in the Supplementary Materials, Figures S5–S8, respectively.

FTIR analysis of hybrid plasma-SKS hydrogel was conducted with a Bruker FTIR spectrophotometer (Bruker, Billerica, MA, USA). For each spectrum, a 512 scan interferogram was collected at single beam absorption mode with a 2 cm^−1^ resolution within the 400–600 cm^−1^ region. For each sample, several FTIR absorption spectra were collected. Five measurements were averaged to obtain the final FTIR absorption spectrum of the sample. Residual water vapor absorption was interactively subtracted from the sample spectra. Spectral calculations were performed by the OPUS (version 4.2) software (Mattson Instruments, Inc., Madison, WI, USA).

#### 2.7.2. Rheological Measurements

Mechanical testing was performed through a rheometry assay. Rheological experiments were performed using a strain-controlled AR-2000ex rheometer (TA Instruments, New Castle, DE, USA) with the hydrogel submerged in MQ water. Briefly, 600 µL cylindrical swollen plasma hydrogels with different amounts of elastin (0, 1, 3 and 5 wt.%) were jellified inside 12 mm diameter. Hydrogel samples were placed between parallel plates of non-porous stainless steel (diameter = 12 mm) and the gap between the plates was adjusted using the minimum normal force that prevent slippage. A gap larger than 1000 µm was always reached after the sample relaxed until equilibrium. Measurements were carried out at 37 °C, with the sample temperature being controlled and maintained using a Peltier device. Several measurements were performed in shear deformation mode. Initially, the range of strain amplitudes over which the hydrogels exhibited a linear region of viscoelasticity (LVR) was determined. To this end, a dynamic strain sweep (with amplitudes ranging between 0.01 and 15%) was carried out at a frequency of 1 Hz to measure the dynamic shear modulus as a function of strain. Secondly, dynamic frequency sweep tests were performed to determine the dependence of the dynamic shear modulus and loss factor on frequency. Specifically, a frequency sweep of between 0.01 and 90 Hz at a fixed strain of 0.3% (corresponding to the LVR hydrogel region) was selected. Evolution of the storage modulus along time was recorded under conditions of 1 Hz and 0.3% of strain in order to evaluate the jellification process. Three replicates of each sample were evaluated. Data were expressed as mean ± standard deviation (SD).

Tensile strength tests were performed using a precision instrument for small-scale testing (ElectroForce 5500 series, TA instrument, New Castle, DE, USA) with a 0.025 N probe. Five 12 mm × 1 mm specimens were clamped using anti-slip clamps leaving a gap of 1.5 mm between the clamps. Tests were performed at 1 mm/s. Load-deformation data were recorded and the force–strain curves of the hydrogels were obtained from the load-deformation data. Young’s modulus was obtained from the slope of the linear section of the force–strain curve before the breaking point and applying the next equation:

E = (F/S)/(ΔL/L) = (F × L)/(S × ΔL)(1)

where E is the Young’s modulus (Pa), σ is the strain (Pa), ε is the stress, S is the area (m^2^), L is the initial length of the sample (m) and ΔL is the elongation (m) and F is the force (N). Data were expressed as mean ± standard deviation (SD) for n = 5.

#### 2.7.3. Characterization of Hydrogel Structure

Morphology of plasma and elastin-plasma hydrogels was investigated using scanning electron microscopy (SEM). In order to maintain the internal structure, hydrogels were dehydrated by a supercritical fluid extraction (SFE) technique based on CO_2_ following the procedure described by Montero et al. [18]. After gelation, during the first 48 h, hydrogels were kept in PBS which was changed every 2 h. Hydrogels were kept 10 days in PBS, where after the first 48 h PBS was changed every 2 days.

Afterwards, hydrogels were dehydrated in graded series of ethanol. Ethanol was further removed using a supercritical reactor Thar R100W (Thar Technologies Inc., Pittsburgh, PA, USA) and gradually replacing ethanol with CO_2_. Subsequently, the temperature and the pressure of the system were raised during 10 min from 23 °C and 60 bar to 35 °C and 100 bars (supercritical point of CO_2_ is 31 °C and 73.96 bar) and left for 90 min under CO_2_ supercritical conditions using a 5 gr/min CO_2_ flow. Then, the system was slowly depressurized to room temperature and atmospheric pressure during 20 min. In order to obtain cross-section micrographs of the dry hydrogels, they were immersed into liquid nitrogen and physically fractured. To increase the conductivity of the sample’s gold, sputtering was performed by using the Leica EM ACE600 (Leica, Wetzlar, Germany). This process is also carried out to protect the samples that otherwise will erode under the electron beam. SEM images were taken with a Phillips XL-30 (F.E.I. Company, Hilsboro, OR, USA), with thermionic emission from a hot tungsten (W) filament. An accelerating voltage of 10 kV was applied in all samples.

### 2.8. Contraction of Hydrogels in Phosphate-Buffered Saline (PBS)

Samples of plasma and plasma with elastin were prepared as described previously (Section 2.4 and Section 2.5). After, their complete gelation, they were immersed in 3 mL of PBS supplemented with 0.01% of sodium azide as preservative and kept under static conditions at 37 °C, 5% CO_2_ pressure and 40% humidity. This solution was changed every 2 days. Contraction studies were determined gravimetrically at different time intervals specifically at 0, 2, 4 and 8 h, on days 1, 2, 3, 5, 7 and 10.

For gravimetrical determination, at appropriated times, PBS was removed, the petri dishes were carefully dried with absorbent paper, to remove the water attached on the petri dish surface and weighed. Afterwards, hydrogels were covered with 3 mL of fresh PBS supplemented with 0.01% of sodium azide and incubated at 37 °C until the next time point. The mass-swelling ratio was calculated from the following equation:

SRw = Mi/Mo(2)

where SRw is the mass swelling ratio, Mi is the mass of the hydrogel at each time point and Mo is the mass of the hydrogel at time zero. In all the experiments a minimum of 3 replicates were measured and averaged.

### 2.9. Cell Studies

#### 2.9.1. Primary Human Keratinocites (hKCs) and Human Fibroblasts (hFBs) Culture

The human fibroblasts (hFBS) and human keratinocytes (hKCs) used for this work were obtained from skin biopsies of healthy donors. All cells used for this work came from a single donor and were obtained from the collection of biological samples of human origin registered in the ‘Registro Nacional de Biobancos para Investigación Biomédica del Instituto de Salud Carlos III’. Previously described methods [61] modified by our laboratory [3,62] were followed for hKCs culture. The growth medium used for hKCs was a 3:1 mixture of Dulbecco’s Modified Eagle Medium (DMEM) (Gibco, Carlsbad, CA, USA) and HAM’S F12 (Gibco, Carlsbad, CA, USA) (hKCs culture medium) containing 10% of fetal bovine serum (FBS), 0.1 nM choleric toxin, 2 nM T3, 5 mg ml−1 insulin, 0.4 mg ml−1 hydrocortisone and 10 ng ml−1EGF (Sigma Aldrich, St Louis, MO, USA) and 1% primocyn. Dulbecco’s modified Eagle’s medium (DMEM) (Gibco, Carlsbad, CA, USA) supplemented with 10% FBS and 1% penicillin/streptomycin was used to culture hFBs at 37 °C in a humidified atmosphere containing 5% CO_2_. Culture medium was regularly changed every 2 days. For all the experiments cells were used between passages 3 and 4.

#### 2.9.2. hFB-Mediated Hydrogel Contraction

Cultured hFBs were encapsulated in plasma and elastin-plasma hydrogels and cell-embedded hydrogel contraction was studied. Cell density was set to 80,000 cells/mL per each replicate of hydrogel. The protocol for hydrogel preparation was slightly modified in order to incorporate hFBs into the hydrogels. Cells were dissolved in 200 µL 0.9% NaCl solution and mixed together with SKS-N3, human plasma (2.3 mg/mL fibrin concentration) and AmchaFibrin. The remaining amount of 0.9% NaCl was mixed together with SKS-Cyclo and 1% CaCl_2_. When dissolved, both components were mixed at 4 °C, placed in 19 mm inner diameter glass vials and incubated at 37 °C, 5% CO_2_ for 1 h to allow the polymerization. Detaching the hydrogels from the glass vials was done with the prewarmed culture medium. Hydrogels were then transferred in a 35 mm diameter petri dish and covered with 3 mL of culture medium and incubated at 37 °C, and 5%CO_2_. Cell-induced hydrogel contraction was studied at specified time intervals (0, 1, 3, 7 and 10 days).

At each time point, hydrogels (free floating in culture medium) were photographed on a dark background along with a ruler which later served as a reference system. To measure the area of hydrogels, photographs were later processed with ImageJ (software v1.52) (ImageJ, U.S. National Institutes of Health, Bethesda, MD, USA). The first measurement (time 0) was taken one hour after the hydrogels were prepared, ensuring complete gelation of every composition. After each time point, hydrogels covered with 3 mL of culture medium, were placed back in the cell incubator. Culture medium was replaced every 2 days to assure adequate cell growth.

#### 2.9.3. Cell Proliferation Assay of Encapsulated hFBs

Cell proliferation of the embedded hFBs was quantified using Alamar Blue ^(R)^ assay (Invitrogen, Carlsbad, CA, USA) after 1, 3, 7 and 14 days. Plasma, elastin-plasma and 3% pure elastin hydrogels, with a volume of 100 µL and 7000 cells encapsulated per hydrogel, were prepared, as previously described in Section 2.9.2, and placed in a 96-well culture plate (Ibidi GmbH^®^, Gräfelfing, Germany). A total of four well plates were used, one for each time point.

After one hour of hydrogel incubation under 5% CO_2_ atmosphere and 37 °C 100 μL of culture medium was added to each well and left undisturbed overnight. At each time point, culture medium was removed and 100 µL of Alamar blue dye (10% Alamar Blue solution in phenol red free and 90% culture medium) was added to each specimen and incubated for 3 h. Subsequently, 75 µL of culture medium from each test sample was transferred to a 96-well plate, and the fluorescence of reduced AlamarBlue^®^ was determined at 530/590 nm excitation/emission wavelengths (Synergy™ HTX Multi-Mode Microplate Reader, BioTek, Winooski, VT, USA). For each time point and sample, a minimum of four replicates were measured, normalized with respect to day 1 and averaged. Data were expressed as mean ± SD.

#### 2.9.4. Cell Viability of Human Keratinocytes (hKCs)

Human keratinocytes (hKCs) viability was determined using MTS Cell Proliferation Colorimetric Assay Kit (MTS assay, BioVision, Milpitas, CA, USA). This procedure quantifies the metabolic activity of keratinocytes seeded on top of the hydrogels by measuring the absorbance of formazan at 490 nm. Absorbance levels are directly proportional to the number of live cells.

Seven samples of 75 µL each, were prepared per composition in a 96-well plate. Hydrogels were prepared following the protocol previously described (without fibroblasts) and incubated under 5% CO_2_ atmosphere at 37 °C for 1 h. Before the cells were seeded on top of the hydrogels, they were covered with 100 μL of culture medium for 4 h allowing excess CaCl_2_ to leach out from the hydrogel, in order to avoid keratinocyte differentiation. Culture medium was then removed, and hKCs suspension containing 1 × 10^4^ hKCs cells in 100 μL of hKCs culture medium was carefully dropped onto the hydrogel surface (except for the negative control). The hydrogels were left undisturbed under 5% CO_2_ atmosphere at 37 °C in an incubator for 2 days to achieve appropriate keratinocytes adhesion to the surface of the hydrogel matrix. After 2 days of incubation, 20 µL of MTS reagent was added to each well and incubated at 37 °C, 5% CO_2_ for 2 h.

Subsequently, 75 µL of hKCs culture medium from each sample was transferred to the remaining empty wells, and formazan absorbance was measured at 490 nm using Synergy™ HTX Multi-Mode Microplate Reader. The whole procedure was done avoiding light exposure as MTS reagent is photosensitive. The cellular viability was calculated from Equation (3):

Relative cell viability = (ODs − OD_B_)/(OD_C_ − OD_B_) × 100 (3)

where OD_S_, OD_B_ and OD_C_ are the optical densities of formazan production for the sample, blank (the corresponding hybrid hydrogel of all compositions (from 0% to 5%) but without seeding keratinocytes on top to subtract the possible interference in the absorbance value of the possible degradation by-product of each hybrid hydrogel) and the control (plasma hydrogel), respectively. ANOVAs were performed respect to plasma hydrogels.

### 2.10. Statistical Analysis and Data Representation

All results were plotted with OriginPro 8.5.1 where data were represented as the mean ± SD from at least three experiments. Statistical analysis was performed using IBM SPSS v.20. Differences between experimental groups were analyzed by using one-way ANOVA with Bonferroni’s posttest. * *p* < 0.05 was considered significant for all statistical tests.

## 3. Results and Discussion

### 3.1. Gelation Time Determination

Hydrogel gelation time for different hybrid plasma-SKS hydrogels (0, 1, 3 and 5 wt.% SKSs content) was determined at 37 °C using the inverted test tube method and rheology (Figure 1b). By the inverted test tube method, plasma gel (0% SKSs content) coagulates in about 18 min (18.16 ± 0.29). However, hybrid-plasma hydrogel showed a noticeable fast-gelling with the increase of SKSs polymeric chains content. Indeed, the incorporation of only 1%SKSs in hybrid hydrogels produce a reduction of almost half of the time observed for plasma control hydrogels (9.5 ± 0.5 min). Thus, hybrid hydrogel with 5% SKSs showed the fastest-gelling with a value in the range of 5 min (5 ± 0.87). As observed from the inverted test tube method and rheology, results showed similar fast-gelling behavior with the increase of SKSs content (Figure 1b), from 33 to 15 min. However, there is no significant difference between plasma control and hybrid hydrogels until the SKSs content reach a percentage above 3%. On the other hand, when evaluated through the inverted test tube method, we show that gelation time for a macroscopic gelation goes from 15 to 5 min for 5 wt.% of elastin content. The discrepancy of values could be explained as the hydrogel as a bulk, even if the matrix is not totally crosslinked, hydrogel is stable enough to retain its shape. Indeed, it is reported that gelation point for plasma hydrogel occurs when only 15% to 20% of the total fibrinogen has been incorporated into the hydrogel [63]. Gelation time of ELRs hydrogels, based on both SKS-N3 and SKS-Cyclo, at 4 °C and at a final recombinamer concentration of 5 wt.% has been previously reported by De la Torre et al. [59] to be in the range of 20 min which is significantly higher. The lower gelation time exhibited by hybrid-plasma-ELRs at 37 °C with respect to 4 °C could be in regard to the fact that the gelation process is produced above the critical temperature solution (Tt) reported for these SKS derivatives (Tt is 38 °C and 29 °C for SKS-N3 and SKS-Cyclo, respectively, see Figure S7A,B).

The presence of ELR polymeric chains does not prevent the plasma gel formation for any of the concentrations in this study. In a similar manner, the orthogonal “click” reaction that takes place between the azide and the cyclooctyne groups present in the two ELR derivatives (SKS-N3 and SKS-Cyclo) is not affected by the presence of the plasma proteins, lipids and multiple platelet growth factors. Visually, at 37 °C and after gelation at the room temperature as well, an increase in the opacity of the gel could be appreciated as well as an off-white tonality with the increase of ELRs content (Figure 1c) which qualitatively could indicate the incorporation of ELRs. Additionally, we performed the test to see the difference in the elasticity of the plasma and hybrid plasma-SKS (Videos S1 and S2), which can also be helpful in proving the presence of elastin in the hydrogel. Plasma and hybrid plasma-SKS (3 wt.% SKSs content) samples were used and each sample was manually stretched so the ability of a hydrogel to resist any permanent change to it, when stress is applied, can be observed. Plasma hydrogel was torn apart after the slightest stress was applied. Hybrid plasma-SKS hydrogel proved its elasticity, it elongated and returned to the original shape and size after applied stress.

### 3.2. Physicochemical Properties of Hybrid Plasma-ELR Hydrogels

The incorporation of ELRs in the hybrid plasma-ELRs hydrogels was confirmed by FTIR. Dried plasma-ELR hydrogels were analyzed by FTIR for the plasma-ELR 5 wt%. From the infrared (IR) spectra (Figure S1) of the formed hydrogels, the peak intensity of azide pendant groups at ~2100 cm^−1^ decreased dramatically, indicating that only very few azide pendant groups existed after the click coupling reaction and that azides were quantitatively transformed into triazoles by reacting with cyclooctyne. In addition, it was reported that very few residual azide pendant groups would not affect biomedical applications [64,65,66].

**Figure 2:**
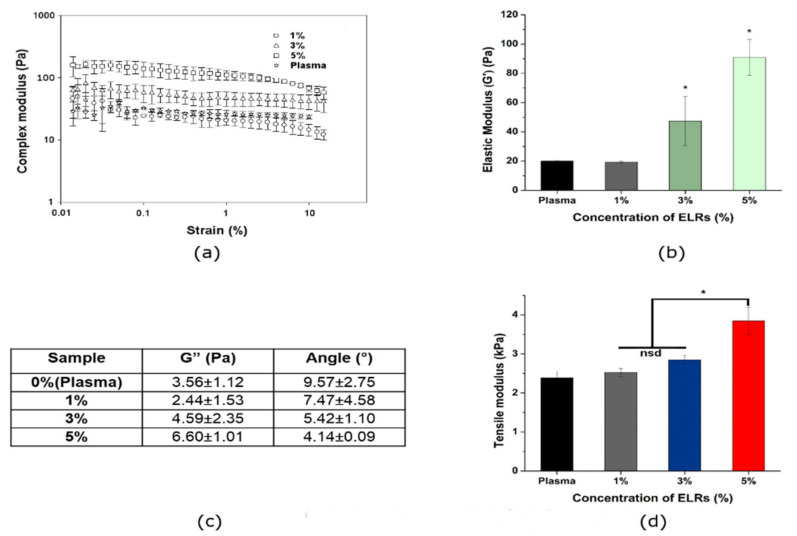
(**a**) Complex modulus of the plasma and hybrid plasma-ELRs hydrogels by strain sweep measurement at 37 °C, (**b**) Elastic modulus (G’) obtained from strain sweep tests of plasma and hybrid plasma-SKSs hydrogels, (**c**) dependence of viscous modulus (G”) and angle on the content of SKSs in the hydrogel composition, (**d**) tensile modulus obtained from strain sweep test of plasma and hybrid plasma-SKSs hydrogels. (* *p* < 0.05).

Dynamic and tensile mechanical properties of hybrid hydrogels were determined by rheology measurement and tensile tests, respectively. The variation of the linear viscoelastic range due to different ELR proportions was measured rheologically. Thus, hybrid-plasma hydrogels containing a constant fibrinogen concentration (1.2 mg/mL) and varying SKSs content (0%, 1%, 3% and 5% in plasma hydrogels) have been rheologically compared. The strain sweep was evaluated in the range 0.01–10% and was found to remain independent of the strain amplitude up to values of about 7–8%. Figure 2a shows the trend in complex modulus for all hydrogels at a frequency of 1 Hz, and at a temperature of 37 °C. Dynamic frequency sweep experiments of all hydrogels provide a gel-like behavior in the frequency range of 0.1 to 1 Hz showed by elastic modulus (G’) values higher than the viscous modulus (G”) (Figure 2a,c) as well as their independence from the frequency (Figure S2). In the case of plasma hydrogels and 1% of SKSs-plasma hydrogels, no significant differences were found with storage moduli values of 20.03 ± 0.49 and 19.33 ± 0.59 Pa, respectively. Similar results for plasma storage moduli, with fibrinogen concentration of 1.2 mg/mL, were reported in the previous work published by our group [18]. Above 1% of SKSs, significant differences appeared as can be observed from the storage moduli values, 47.39 ± 16.78 and 90.93 ± 12.36 for 3% and 5% of SKSs, respectively (Figure 2b). Values for angle were recorded for the four conditions (0%, 1%, 3% and 5% SKSs) and a clear decrease of the values as function of SKS percentage was observed as can be seen in Figure 2c which indicates the improvement of the elastic recovery properties of plasma-SKS systems [67,68].

In a similar way to G’, we observed that Young modulus values followed an increasing trend depending on the amount of SKSs in our hydrogels, as can be seen in Figure 2d. No significant differences can be found between 1% and 3%, however, significant differences were observed for 1 and 3 with respect to 5%.

Although an increase in the Young modulus value could be observed in the 0% and 1% samples, no significant difference can be appreciated among these two types of samples. As described in literature [69] a full IPN increase the mechanical properties is related to the crosslink degree. In the present research, the degree of crosslinking was modified by increasing the concentration of SKS. It is important to mention that the increase of SKS content mainly contributed to the improvement of mechanical properties. In the literature, pure elastin like hydrogel show under similar test conditions a complex shear modulus in the same range [70].

In order to investigate the internal morphology of elastin-plasma hydrogels and compare it to the morphology of plasma hydrogels, SEM was performed. The aim was to obtain a comprehensive idea on how ELRs could affect the density of the hydrogel scaffold. Generally speaking, a higher concentration of elastin is expected to form denser and compact scaffolds which also affects mechanical properties.

**Figure 3:**
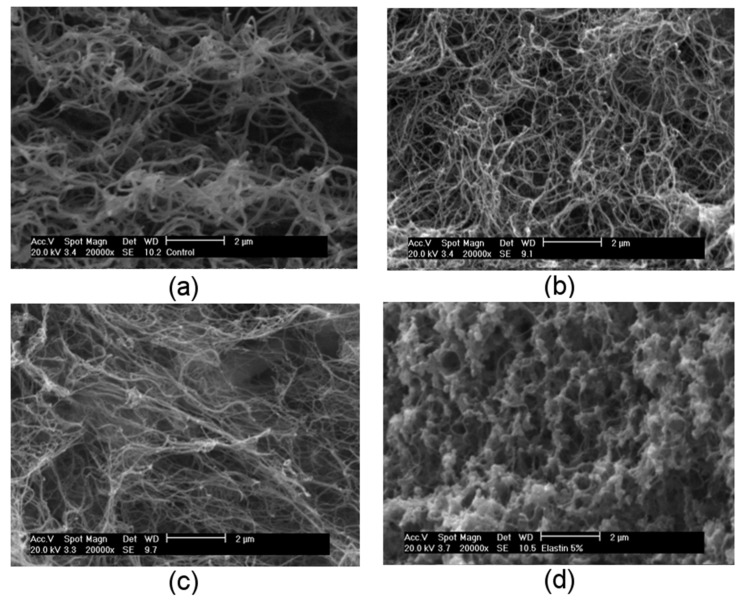
Scanning electron microscopy (SEM) images of (**a**) plasma and elastin-plasma hydrogels with (**b**) 1%, (**c**) 3% and (**d**) 5% elastin concentration after 10 days of incubation in PBS at 37 °C, at 20,000× magnification and an accelerating voltage of 10 kV. Scale bar corresponds to 2 µm.

As mentioned before, plasma hydrogel is formed during the physiological coagulation cascade where fibrinogen goes through thrombin-mediated cleavage in the presence of Ca^2+^ ions. SEM micrographs of plasma and elastin-plasma hydrogels after 10 days of incubation in PBS are shown in Figure 3. Plasma hydrogels, as expected, showed homogeneous and delineated fibrillar structure with high interconnectivity and porous environment. Apart from fibrinogen, additional plasma proteins, such as albumin, globulins, etc., are embedded in the hydrogels during the polymerization process of fibrinogen. However, the presence of those proteins usually provides stiffer and irregular fibers which are not appreciated in Figure 3a. It has been previously reported in literature [18] that after 7 days of incubation in PBS at 37 °C almost 90% of initial plasma proteins are released from the hydrogels. Since these micrographs were captured after 10 days of incubation in PBS at 37 °C we can assume that most of the initial plasma proteins have leached out from the hydrogel leaving them with a less stiff structure and more regular fiber formation.

From SEM micrographs, it is clear that plasma and elastin-plasma hydrogels have different morphologies. For the lowest SKSs concentration, Figure 3b, as in plasma hydrogels, a homogenous and delineated fibrillar structure with high interconnectivity and porous environment is observed. High interconnectivity of fibers within hydrogels provides evident advantages in relation to tissue engineering. The clear fibrillar network for plasma and plasma with 1% and 3% elastin (Figure 3a–c) proves that the conversion of fibrinogen to fibrin occurred [71,72]. It also leads to the conclusion that existence of fibrin in SKSs cross-linking mixture is enough for the formation of IPN. In Figure 3, it can also be observed how the structure becomes denser, less delineated, and compact with the increase of elastin concentration.

Hydrogels with the highest elastin concentration, Figure 3d, present an intricate structure with more irregular and less-defined fibers. Fibrin fibers can still be observed (fibril fibers are indicated by arrows → ← in Figure 3d), but they are coated with elastin. A more dense and globular structure, seen in the SEM micrographs for 5% elastin (Figure 3d), can be explained by the different assembly behavior of elastin in a function of the gelation temperature. Thermal sensitivity of ELRs is characterized by transition temperature (T_t_) which is associated with the conformational reorganization on the molecular level. In the completely reversible process, below T_t_ polymer chains are soluble in water, whereas above T_t_ they self-assemble into nano and microaggregates and become insoluble [73]. Transition temperature of ELRs used in this work is reported to be Tt 38 °C (SKS-azide) and 29 °C (SKS-cyclo) (Figure S7). Knowing that the gelation temperature of plasma and elastin-plasma hydrogels used in this work was 37 °C, one can conclude that the gelation for these hydrogels happened above T_t_. As mentioned before, above T_t_ ELRs have a tendency to self-assemble into nano and microaggregates adopting globular structures which explains the spherical arrangement of 5% elastin.

Comparison of the plasma and elastin-plasma hydrogels microstructure in term of fibrin fiber diameter was analyzed by ImageJ (see fiber diameter histogram in Supplementary Materials (Figure S3a)) and the analysis is shown in Figure S3b. Plasma gels showed a fiber diameter of 154.70 ± 31.40 nm. When increasing the content of ELRs in the hybrid hydrogels, a reduction of the average fibrin fiber diameter was observed. Thus, for a content of 1% ELRs, an average fiber diameter gets reduced to values in the range of 111.84 ± 26.38 nm whereas for 3% ELRs fiber diameter is in the range 89.38 ± 25.14 nm.

After the enzymatic cleavage of fibrinopeptides by thrombin, fibrin monomers aggregate forming the so called two-stranded protofibrils which aggregates laterally to produce fibers [74]. Density of protofibrils connections strongly decreases with the increase in fiber diameter meaning that thin fibers have more strongly connected protofibrils than thick fibers [75]. Fiber aggregations are affected by several factors that can provide different fiber size. Those factors include thrombin, fibrinogen or calcium concentration, factor XIIIa, the ionic and pH conditions and the presence of additional proteins [76,77,78,79]. Thus, the presence of elastin protein could affect the mechanism of fibrin fibers formation. A similar effect has been reported by Lai et al. where the presence of collagen in fibrin gels affects the average fiber diameter [80].

Li et.al. [75] studied fiber diameter of fibrin fibers and its impact on the fiber stiffness. They reported that “thin fibers can be 100 times stiffer than thick fibers”. In other words, they proved that increase in fiber diameter drastically decreases elastic modulus. Reduction of the average fibrin fiber diameter can be observed in Figure S3b. and Figure 2b shows how elastic modulus strongly increases with the increase of elastin concentration. With these results we can confirm that with the increase of elastin concentration fiber diameter is decreased and increase in hydrogel elasticity and mechanical properties is observed.

### 3.3. Contraction of Hydrogels in Phosphate-Buffered Saline (PBS)

Plasma hydrogels suffer from weak mechanical properties and they have a tendency to shrink during culture, transportation and implantation [18,19,20,81,82]. That limits its translational success when used in skin tissue engineering applications, such as the production of in vitro skin substitutes. Despite the progress in this field, there is still a need to develop new strategies in order to overcome the aforementioned limitations. This contraction is described to be mediated by the tension that fibroblasts are able to induce during prolonged culture producing a lattices contraction of the matrix [20,83,84]. In the recent study Montero et al. [18] demonstrated, for plasma gels, that this contraction phenomenon takes place also in the absence of cells, so additional factors play an important role.

Thus, the effect of the reinforced plasma hydrogels by the presence of SKSs network has been studied in terms of contraction. Contraction was analyzed in the absence of cells and measured by deswelling behavior (assessed by mass ratio) of every hydrogel composition, incubated at 37 °C in 3 mL PBS supplemented with 0.01% sodium azide for 10 days.

**Figure 4:**
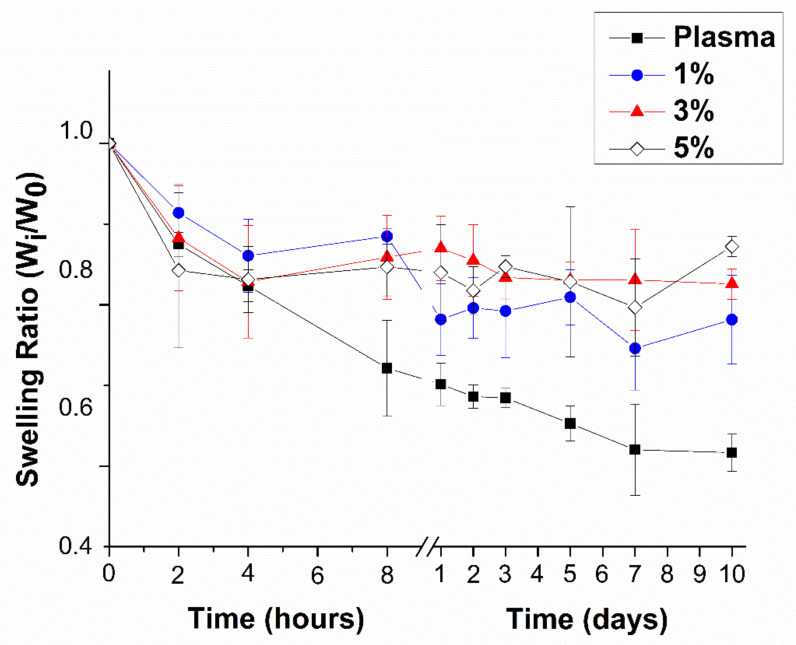
Plasma and hybrid plasma-SKSs contraction, determined as mass loss for 10 days, in PBS, pH 7.4, at 37 °C for each gel composition (n = 3). Data reported as mean ± SD. Percentages indicate % *w*/*v* elastin content in hybrid hydrogels.

The addition of an SKSs network to the plasma-based hydrogels had an impact on the deswelling behaviour as can be observed in Figure 4. The obtained results, for all hydrogel compositions, showed that they contract after incubation in aqueous environment at 37 °C. None of the hydrogels showed swelling behaviour; the opposite, contraction behaviour, is observed. Analyzing the results, it can be observed that contraction decrease strongly when adding at least 1% *w*/*v* of elastin to the hydrogel composition. Plasma hydrogels showed fast contraction during the initial 8 h, showing contraction of 27.88 ± 5.950%. On the other hand, hydrogels with 1%, 3% and 5% ELRs showed reduced tendency to deswell. They showed contraction of only 1.82–2.42 fold decrease in comparison to plasma hydrogels, which demonstrates the reduction in contraction. However, the increase in SKSs content from 1% to 3% and 5% does not produce additional significant reduction in contraction. The contraction of SKSs 1% at 8 h was 11.51 ± 1.02% while the increase of SKSs to 3% and 5% produced a similar reduction, being 14.10 ± 5.20% and 15.30 ± 3.60%, respectively.

We can say that the deswelling takes place in two steps: (1) fast deswelling step which occurs during the first 8 h and (2) a second step, after the initial 24 h, where the deswelling is slower. After 10 days, plasma and 1% elastin plasma hydrogels showed a more significant decrease in contraction, 38.34 ± 2.29 and 21.85 ± 5.49, respectively. For the hydrogels with 3% and 5% SKSs contraction still happens but in reduced amounts. Quite surprisingly for 3% elastin plasma hydrogel, after 3 days in aqueous solution contraction seems to slightly increase, having the final contraction after 10 days of 17.45 ± 1.88. For 5% elastin plasma samples, after 10 days of incubation in PBS, contraction continues to follow a decreasing trend reaching 12.77 ± 1.22. During the first 4 h significant differences between plasma and hybrid-plasma hydrogels were not found. For longer incubation time (>8 h until 10 days) significant differences were observed for 3% and 5% elastin plasma hydrogels with respect to plasma.

It seems that for long-term stability (10 days) there is a significant effect in the contraction behaviour with an increase of SKSs content. After the initial 8 h, values for all 3 SKSs compositions were quite similar while after 10 days the lowest contraction was observed for 5% elastin plasma hydrogel.

We can say that incorporation of SKSs positively affected hydrogel contraction. This could be explained with elastin ability to retain water. SKS ELRs contain serine (S) amino acids in their sequence: [(VPG**S**G-VPGKG-VPG**S**G)_2_-RGD-(VPG**S**G-VPGKG-VPG**S**G)_2_]_6_ which increases the transition temperature of the polymer, allowing the retention of a higher amount of water at 37 °C. Therefore, at room temperature, polymer will not transition, it will retain higher amounts of water and increase transparency [85]. Moreover, the incorporation of an IPN increases crosslinking density, which aids in the retention of liquid.

### 3.4. Cell Studies or Biological Evaluation

#### 3.4.1. hFBs-Mediated Gel Contraction

The ability of hFBs cells to contract plasma and hybrid plasma-ELRs hydrogels was studied following the floating lattice model [86]. where contraction is reflected by the reduction of hydrogel diameter (XY plane contraction) [87].

**Figure 5:**
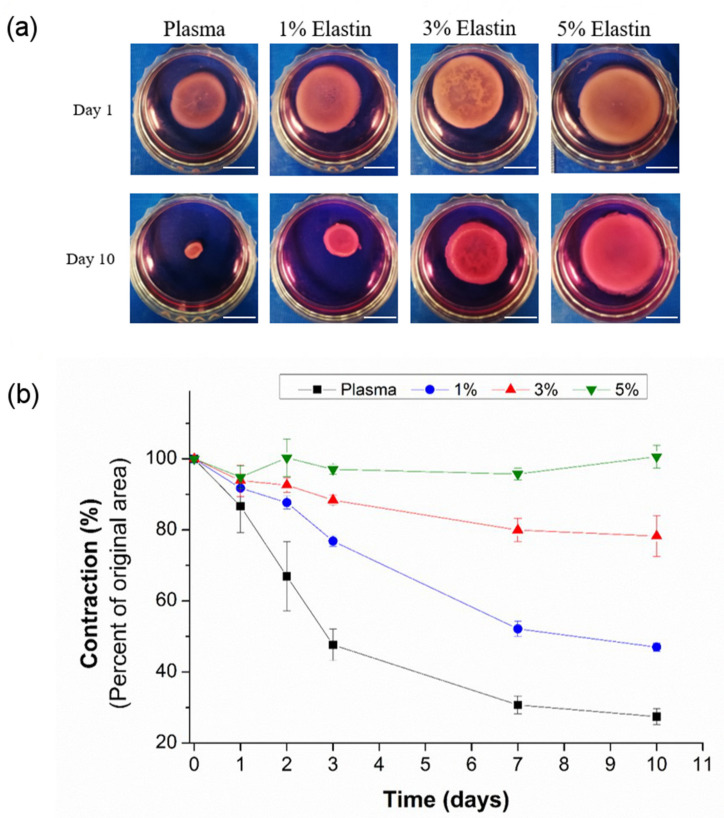
(**a**) Images of each gel composition in culture medium at time points 1 and 10 days. Percentages indicate % of elastin in elastin-plasma hydrogels. The scale bars correspond to 1 cm. (**b**) Cell-induced hydrogel contraction ratio for each hydrogel composition (n = 3), in culture medium at 37 °C. Data reported as mean ± SD. Percentages indicate % *w*/*v* elastin content in plasma hydrogels.

Contractions can be directly observed (Figure 5a) by comparing the hydrogels from day 1 to day 10 of incubation. After 10 days a significant decrease in the diameter of the plasma hydrogel can be noticed, as well as loss in its roundness (see Figure S4). Similar behaviour is present with 1% elastin-plasma hydrogel where diameter drastically decreased between day 3 and day 7 but the roundness of hydrogel is maintained (Figure S4). Contrarily, hydrogels containing high concentrations of elastin (3% and 5%) do not present an appreciable loss in diameter even after 10 days of incubation. Images from Figure 5a, analyzed in terms of roundness by Image J (Figure S4), clearly show how these hydrogels maintained their shape and circular structure.

Hydrogel’s contraction was clearly affected by the incorporation of elastin into plasma hydrogels. This effect can be observed from the Figure 5b, where the contraction is presented as a percentage of the original area. Significant inhibition of the hydrogel contraction, promoted by human dermal fibroblasts, in respect to plasma hydrogels, was found for all elastin-plasma hydrogel compositions (*p* < 0.05). This significant difference is observed in all time points except for time point “day 1”.

After 2 days of incubation, elastin-plasma hydrogels containing 1% of elastin, partially inhibited the contraction in respect to plasma hydrogels. Thus, the incorporation of 1% SKSs provides a partial inhibition of the contraction with values of only 87.70 ± 1.81%, while plasma alone showed a contraction to 66.91 ± 9.68%. However, when increasing the SKSs content to 3% and 5%, the contraction was much lower (92.63 ± 2.12) for 3% elastin and for 5% elastin almost no contraction occurred (100.30 ± 5.28). Furthermore, after 10 days, contraction is drastically reduced in hydrogels with 3% (78.25 ± 5.78) and 5% (100.62 ± 3.18) elastin content compared to plasma hydrogels (27.44 ± 2.21). The results indicate that the incorporation of SKSs inhibits contraction in a dose-dependent manner.

The contraction inhibition in the hybrid hydrogels could be due to the matrix stabilization provided by the presence of the SKSs second network in the IPN hydrogels. This second network is able to reinforce the matrix not only by increasing the mechanical properties, as previously shown, but also by its degradation stability. Fibrin degradation promoted by fibroblasts and cell-mediated contraction are the main reasons for plasma hydrogel contraction [20]. Unlike plasma hydrogels, elastin plasma hydrogels showed excellent matrix stability even at a longer time which could be attributed to the fact that native ELRs hydrogels present high stability in vivo. Indeed, this long-term stability in vivo has been previously demonstrated by subcutaneous ELR hydrogel injection at the hypodermis of mice [40].

As shown from SEM micrographs, plasma and elastin-plasma hydrogels have different morphologies. Homogeneous, delineated, fibrillar structure with high interconnectivity and porous environment is observed for plasma and 1% elastin-plasma hydrogels. Their morphologies can be used to explain the change in their diameter/roundness. Plasma hydrogels have a porous environment, and cell growth is observed after day 3 which results in loss of roundness. By contrast, despite good proliferation results, for 1% elastin-plasma hydrogel, the round shape is maintained, whereas for 3% and 5% elastin-plasma hydrogels the structure becomes denser, less delineated, compact and less porous. This confirms why they retain their round shape better. Since cell proliferation in less porous environment is not good, cells are not pulling the hydrogel matrix, thus the round shape is retained.

#### 3.4.2. Cell Proliferation of Encapsulated hFBs

**Figure 6:**
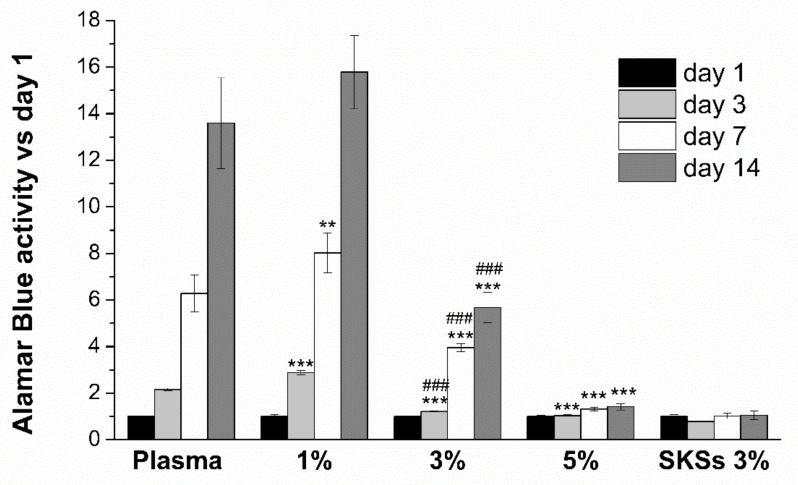
Alamar Blue cell proliferation for encapsulated human fibroblasts in plasma, hybrid plasma-SKSs (1%, 3% and 5%) and SKSs (3%) hydrogels over time (1, 3, 7 and 14 days) normalized respect to day 1 values. Data are reported as mean ± SD (n = 4). ANOVAs were performed for 1, 3 and 5% hybrid plasma-SKSs hydrogels respect to plasma samples at each time point (** *p* < 0.01 and *** *p* < 0.001) and for plasma-SKS 3% respect to pure elastin hydrogel (3%) at each time point at significance level of (### *p* < 0.001).

Proliferation of encapsulated hFBs in plasma, all elastin-plasma (1, 3 and 5%) and 3% pure elastin hydrogels was evaluated by AlamarBlue^®^ assay at different cell culture time points (1, 3, 7 and 14 days). The results in Figure 6 show normalized signal values with respect to hydrogels at day 1. The incorporation of SKSs in the plasma hydrogels for a concentration equal or lower than 1% provides a significant enhancement of cell proliferation in comparison to plasma hydrogels for days 3 and 7. However, when the concentration of elastin in the hydrogel increases to 3% or above, cell proliferation is compromised. In fact, while plasma and 1% hybrid hydrogels show an increase in cell proliferation from day 1 until 14 days, 3% and 5% show a limiting increase in cell proliferation with time and non-proliferation, respectively. Indeed, 5% hybrid hydrogels show the same viability as 3% pure elastin. This indicates that it is possible the loss of porosity and the inability to degrade elastin limits the ability of the cells to reshape their environment and proliferate. On the other hand, when elastin is present in a low percentage, it stimulates cell viability. This effect may be due to the presence of RGD sequences in the elastin chain and a constriction inside the hydrogel similar to that of the controls.

Additionally, it is worth mentioning the enhancement in cell proliferation at any time point shown by hybrid hydrogels with 3% SKSs content in respect to pure elastin hydrogels. This result may be due to the supportive microenvironment provided by the fibrin matrix for cell attachment and proliferation coming from the RGD presence in fibrinogen which support cell attachment.

The increase in cell proliferation at 14 days in the case of plasma and 1% hybrid hydrogels it could be due to possible degradation of the fibrin network by enzymes secreted by the cells, leaving space for hFB spreading and migration. This effect is not appreciated for a hybrid system with 3% and 5% SKSs content because the non-susceptibility to degradation of ELRs matrices.

Knowing that SKS amino acid sequence contains an RGD cell adhesion sequence and that incorporation of elastin in 3D hydrogels increases cell adhesion [88], an increase in cell proliferation was expected as elastin was incorporated in the fibrin scaffolds. However, this does not correlate with the decrease in cell proliferation as elastin concentration is increased to 3% and 5% in plasma-elastin hydrogels.

This behaviour could be explained with the increase of crosslinking degree and decrease in hydrogel porosity. This was observed from the SEM micrographs, which were also correlated with the rheology results. hFBs have difficulties spreading inside more compact crosslinked environments such as hybrid plasma-ELRs matrices and that remodeling takes longer than usual [89]. It was shown how an increase of crosslinking degree increases the stiffness of the environment, causing limitations for cell elongation, migration and proliferation [90].

Other studies have shown that low porosity values negatively affect cell adhesion and proliferation [91]. All mentioned above could be used to explain how the incorporation of elastin increases cell proliferation compared to plasma-based hydrogels, but a decrease in hFBs proliferation can be observed when elastin concentration is increased in plasma-elastin hydrogels. Moreover, plasma proteins are known to regulate cell proliferation, gene expression, survival and motility [92]. This could be one of the reasons why fibroblasts do not proliferate as expected in 3% elastin gels, which are also supposed to present high crosslinking degree and low porosity, therefore hindering hFBs adhesion and proliferation.

#### 3.4.3. Cell Viability of hKCs

**Figure 7:**
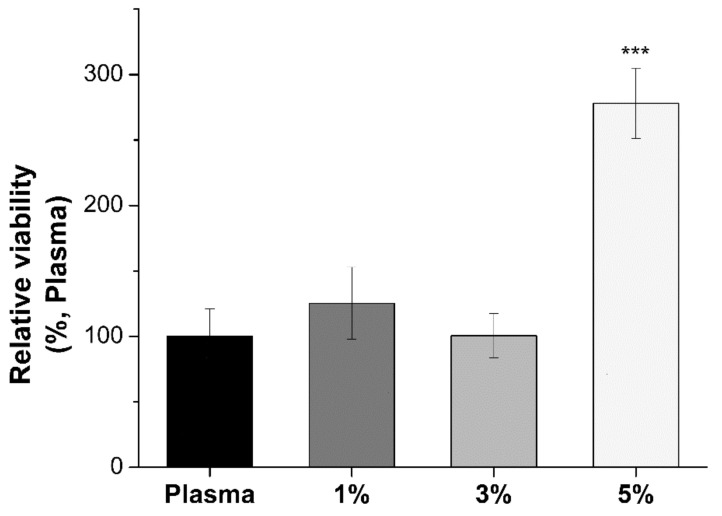
Relative viability of keratinocytes in plasma and hybrids hydrogels by MTS assay at day 2. Data are reported as mean ± SD (n = 4). ANOVA analysis were performed for 1, 3 and 5% hybrid plasma-SKS hydrogels respect to plasma samples (**** *p* < 0.001).

The idea behind this work resides in a possible application of the present system to use as a bilayered organotypic skin in vitro. In order to make this skin model, two types of cellular components are required. Apart from hFBs, which are found in the proposed model, a second cellular component is required, hKCs. They were seeded on top of the hydrogels and their attachment capacity was determined in function of the SKSs content on the hydrogels. In other words, the effect of the interpenetrating biodegradable SKSs network on primary hKCs was studied. An MTS assay was performed on day 2 after seeding the hKCs onto the hydrogels (Figure 7).

The results showed how the metabolic output from hKCs within hydrogels containing 1 and 3% SKSs is not significantly different from plasma hydrogels. For these two SKSs concentrations we can say that the hKCs proliferated almost at the same level as on plasma hydrogels. On the other hand, an increase in hKCs proliferation is observed for hydrogels containing 5% SKSs and they showed a significant difference in proliferation in comparison to plasma hydrogels. This phenomenon may be explained using the fact that an increase of SKSs content provides a stiffer environment which is well known to affect cell adhesion, spreading and proliferation [93].

Plasma-based hydrogels are expected to have good adhesion performance due to the presence of fibrin [2,12]. Increasing percentages of ELRs are also supposed to be good for cell proliferation providing a supportive non-degrading network. In addition, as mentioned before, ELRs contain an RGD component, a sequence known to promote cell adhesion [88].

## 4. Conclusions

Dual crosslinked networks based on plasma-derived fibrin with different ELRs (SKS-N3 and SKS-Cyclo) content were successfully obtained with the purpose of addressing the current limitations of the existing plasma-derived fibrin hydrogels for the generation of dermo-epidermal equivalents. Mechanical properties of these novel skin tissue constructs, as determined in tension and oscillatory shear, showed a significant improvement when SKSs content is equal or higher than 3%. This fact could be attributed to the formation of a full IPN, consisting of both the fibrin network and the second network via click reaction between azide- and cyclooctyne-modified ELRs. Indeed, a denser and compact internal morphology is observed with the increase of SKS content which could be responsible for this mechanical improvement. This superior behavior is also reflected in terms of shrinkage, both in the absence and presence of cells, where an increase of scaffold stability was observed with the increase of SKS content. However, hFBs 3D viability showed the need to achieve a balance. An extremely dense matrix, corresponding to the highest SKS content, showed a reduction in hFBs proliferation while the lowest SKS content (1%) showed an improvement in cell proliferation over time with respect to plasma hydrogels. By contrast, high biocompatibility supporting hKCs on their surface was observed for all the SKS systems, independent of the percentage. Thus, hybrid-plasma hydrogels with an SKS content in the range of 1–3% emerge as the best candidates for application in skin tissue engineering. In the near future, in vitro studies will be carried out to evaluate their ability to form dermo-epidermal equivalents.

## Data Availability

The data presented in this study are available in Supplementary Materials or upon request from authors.

## Supplementary Materials

The following are available online at https://www.mdpi.com/article/10.3390/polym13132114/s1, Figure S1. Fourier-transform infrared (FTIR) spectra of (A) SKS-N3 and (B) hybrid plasma-SKS gel (5%). Each curve is representative of five measured spectra; Figure S2. (A) Storage and (B) loss modulus of the plasma and hybrid plasma-SKSs hydrogels by dynamic Frequency sweep at a fix strain of 0.3% and 37 °C; Figure S3. (A)Fiber diameter histogram of the plasma and hybrid plasma-SKSs hydrogels with 1, 3 and 5% content of ELRs obtained by the corresponding SEM figures (from Figure 3A); (B) Average fiber diameter of the fibrin fibers as a function of SKSs hydrogel content (data expressed as mean ± SD, n = 32 and ANOVA results performed for each hydrogel with respect to plasma samples at significant level of *** *p* < 0.005; Figure S4. Roundness of each gel composition incubated at 37 in complete DMEM at different time points. Roundness was determined by Image J (Roundness = 4 × Area/(π × Major Axis^2^); Figure S5. ^1^H-RMN for SKS-Cyclo (A), and SKS-N3 (B). Both in DMSO-d6; Figure S6. FTIR for SKS-Cyclo (A) and SKS-N3 (B); Figure S7. DSC for SKS-Cyclo (A), Tt = 29.43 °C and SKS-N3 (B), Tt = 38.76 °C; Figure S8. Matrix-Assisted Laser Desorption/Ionization Time of Flight Analysis. Video S1: Plasma hydrogels elasticity. Video S2: Plasma-SKS (3 wt.% SKSs content) elasticity.
