# Supplementary material for: Elastin-Plasma Hybrid Hydrogels for Skin Tissue Engineering
Source: Polymers (Basel). 2021 Jun 28;13(13):2114. doi: 10.3390/polym13132114 (PMC8271496; doi:10.3390/polym13132114)
Supplement: Supplementary file 1 [file polymers-13-02114-s001.zip › polymers-1255706-supplementary.pdf]

polymers

MDPI

Supplementary Materials

Elastin-Plasma Hybrid Hydrogels for Skin Tissue Engineering

Marija Stojic [1,2], Joaquín Ródenas-Rochina [3] María Luisa López-Donaire [2], Israel González de Torre [4, *], Miguel González Pérez [4], José-Carlos Rodríguez-Cabello [4], Lucy Vojtová [1], José Luis Jorcano [2] and Diego Velasco [2,5,*]

[1] CEITEC-Central European Institute of Technology, Brno University of Technology, Purkyňova 123, 61200 Brno, Czech Republic;
[2] Department of Bioengineering & Aerospace Engineering, University Carlos III of Madrid (UC3M), Avda. Universidad, 30. 28911 Leganes, Madrid, Spain;
[3] Centre for Biomaterials and Tissue Engineering, CBIT, Universitat Politecnica de Valencia, Camino de Vera s/n. 46022, Valencia, Spain; (email of Joaquin) (J.R.-R)
[4] BIOFORGE (Group for Advanced Materials and Nanobiotechnology), University of Valladolid, CIBER-BBN, 47011 Valladolid, Spain
[5] Instituto de Investigación Sanitaria Gregorio Marañón, Madrid, Spain
* Correspondence: divelasc@ing.uc3m.es (D.V.-B.), igonzalez@bioforge.uva.es (I.G.T) (I.G.T)

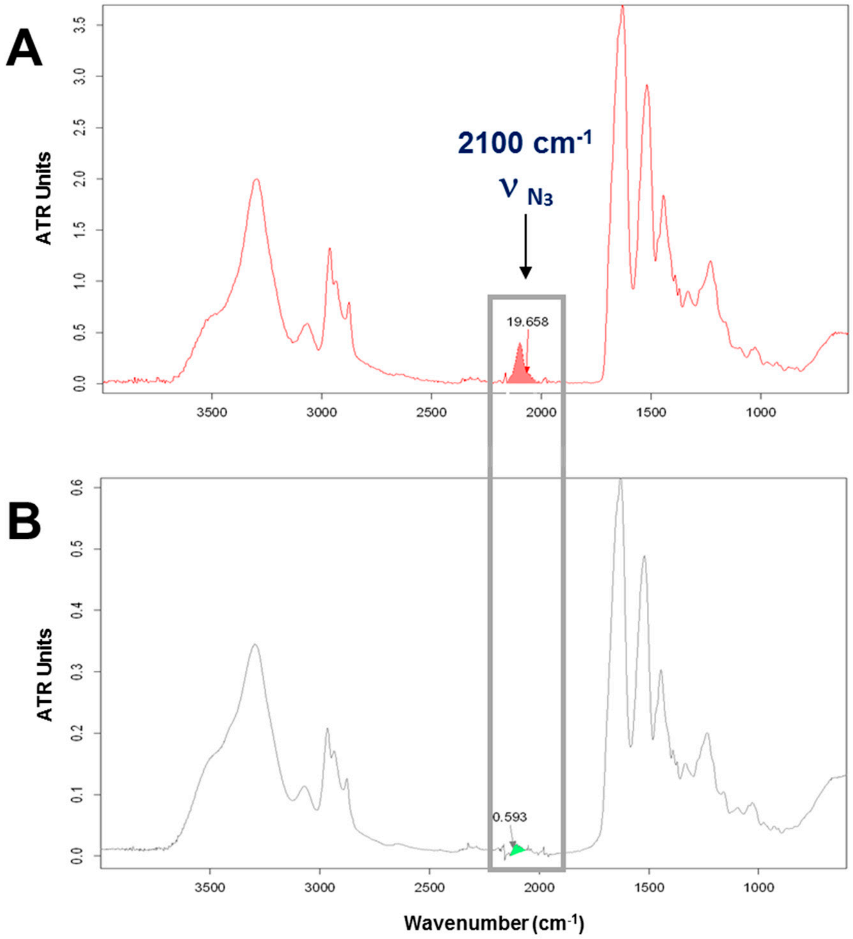

Figure S1. FTIR spectra of (**A**) SKS-N_3 and (**B**) hybrid plasma-SKS gel (5%). Each curve is representative of five measured spectra. Taking these curves as representative as well as the integral values of the peaks (normalized to the values of the highest signal) and considering that each SKS-N_3 molecule has 24 Lysine

residues, we can see that less than one lysine (concretely 0.7) remains uncross-linked in the hybrid plasma-SKS gel.

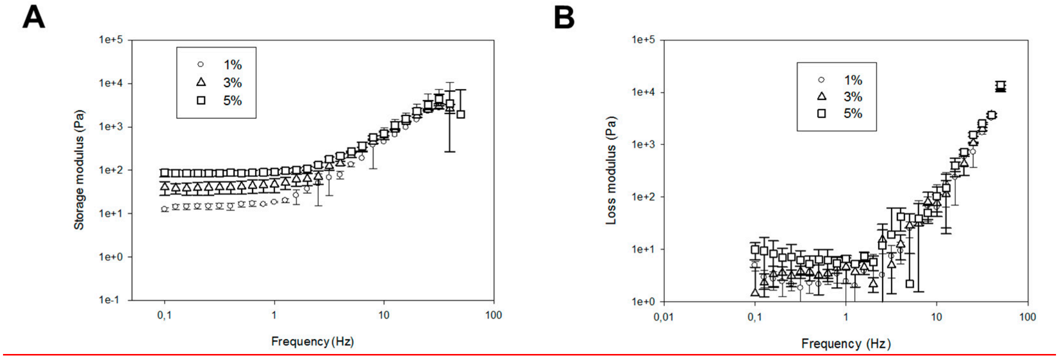

Figure S2. (**A**) Storage and (**B**) loss modulus of the plasma and hybrid plasma-SKSs hydrogels by dynamic Frequency sweep at a fix strain of 0.3% and 37 °C.

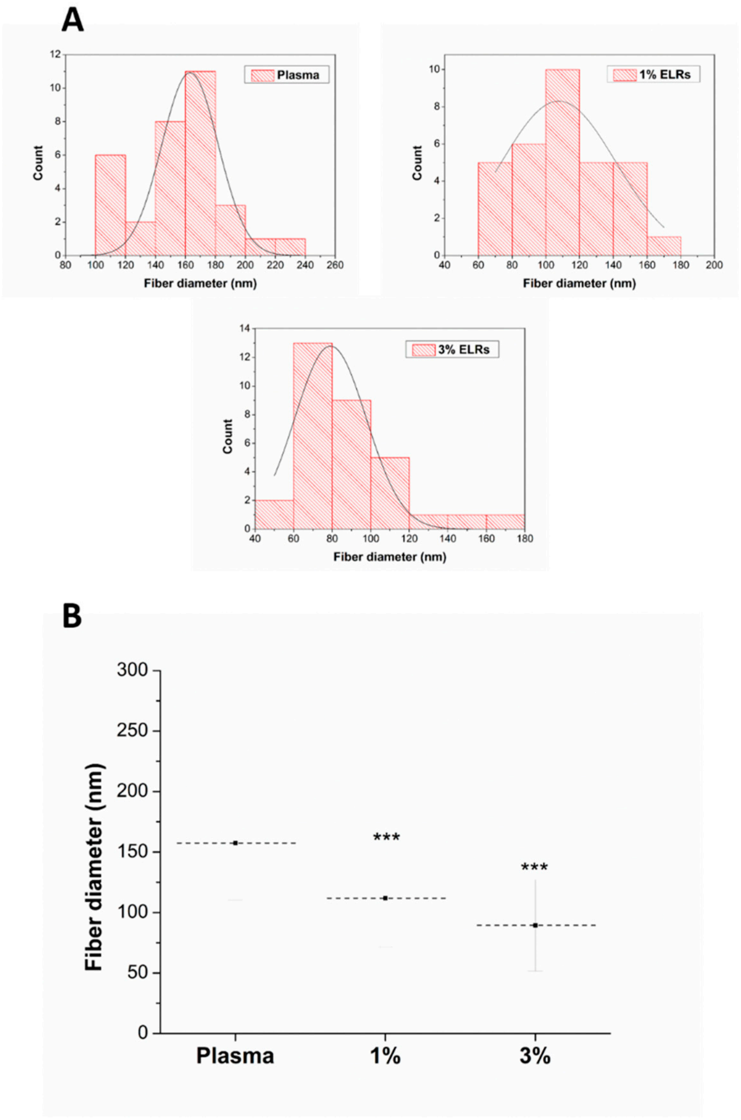

Figure S3. (**A**)Fiber diameter histogram of the plasma and hybrid plasma-SKSs hydrogels with 1,3 and 5% content of ELRs obtained by the corresponding SEM figures (from Figure 3A); (**B**)

Average fiber diameter of the fibrin fibers as a function of SKSs hydrogel content (data expressed as mean ±SD, n=32 and ANOVA results performed for each hydrogel with respect to plasma samples at significant level of *** $p < 0.005$.

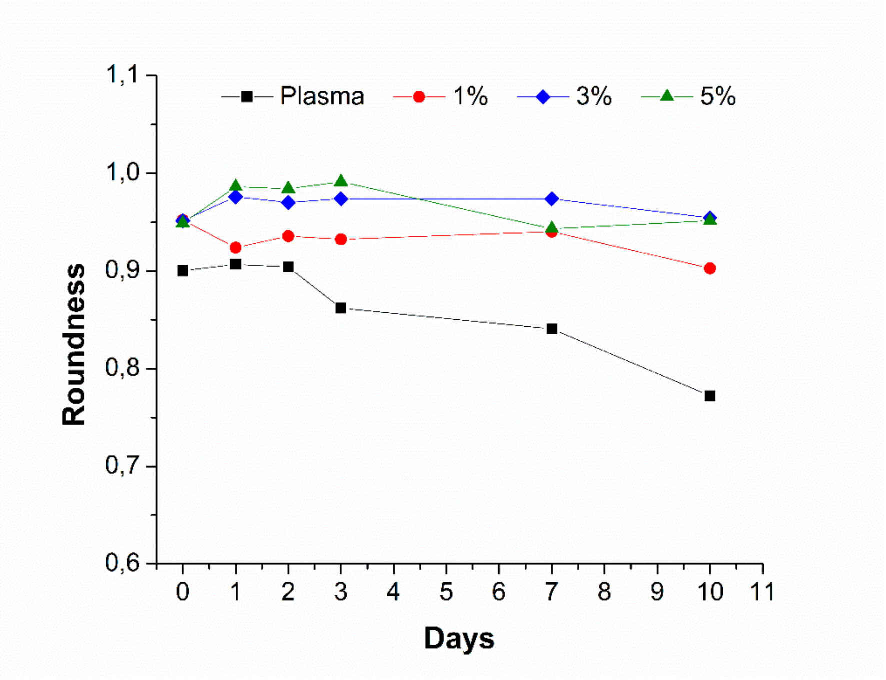

Figure S4. Roundness of each gel composition incubated at 37 in complete DMEM at different time points. Roundness was determined by Image J (Roundness = 4 × Area/ (π × Major Axis [2]).

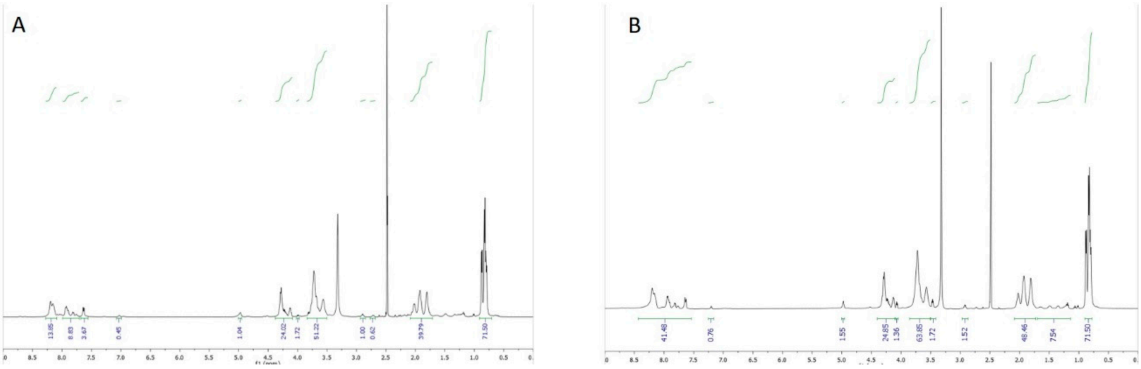

Figure S5. 1H-NMR for SKS-Cyclo (**A**), and SKS-N_3 (**B**). Both in DMSO-d6.

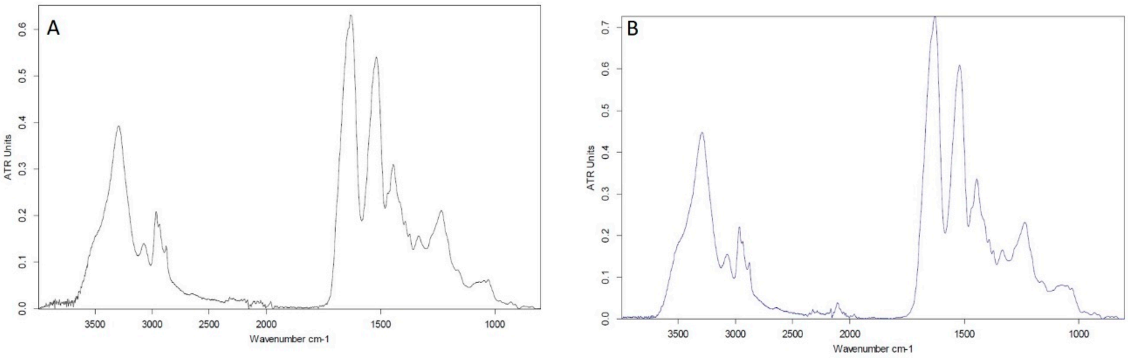

Figure S6. FTIR for SKS-Cyclo (**A**) and SKS-N_3 (**B**).

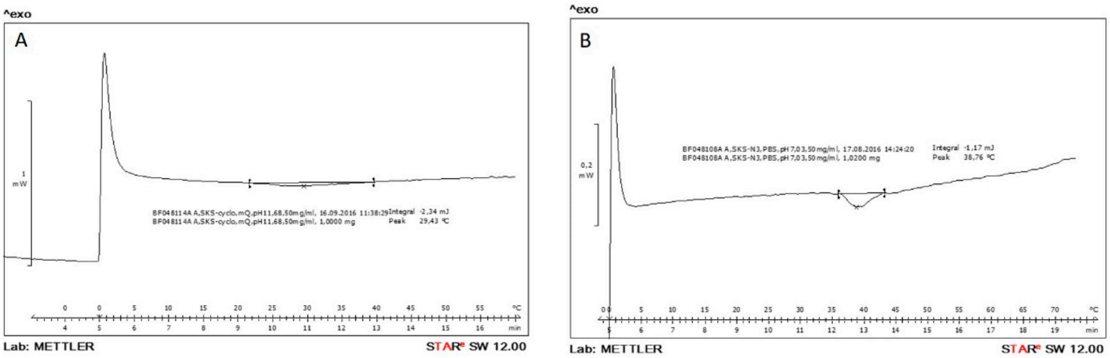

Figure S7. DSC for SKS-Cyclo (**A**), Tt=29,43°C and SKS-N_3 (**B**), Tt=38,76°C.

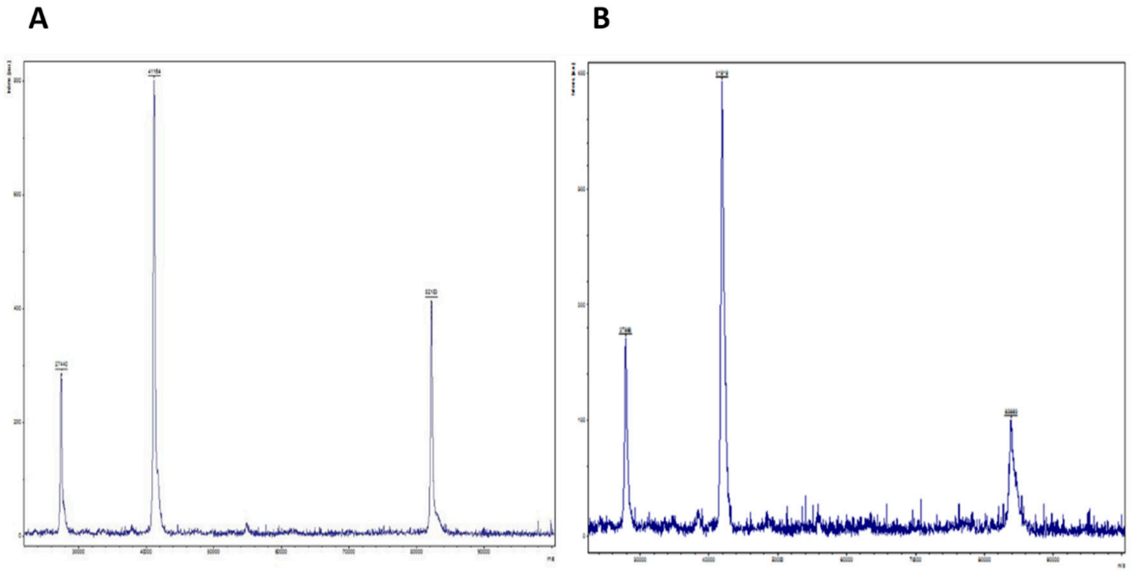

Figure S8. MALDI-ToF for SKS-Cyclo (**A**) and SKS-N_3 (**B**).
